# Apparent sixth sense in theropod evolution: The making of a Cretaceous weathervane

**DOI:** 10.1371/journal.pone.0187064

**Published:** 2017-11-02

**Authors:** Bruce M. Rothschild, Virginia Naples

**Affiliations:** 1 West Virginia University College of Medicine, Department of Medicine, Morgantown, West Virginia United States of America; 2 Carnegie Museum, Pittsburgh, Pennsylvania, United States of America; 3 Northern Illinois University, DeKalb, Illinois, United States of America; State Museum of Natural History, GERMANY

## Abstract

**Objective:**

Two separate and distinctive skills are necessary to find prey: Detection of its presence and determination of its location. Surface microscopy of the dentary of albertosaurines revealed a previously undescribed sensory modification, as will be described here. While dentary “foramina” were previously thought to contain tactile sensory organs, the potential function of this theropod modification as a unique localizing system is explored in this study.

**Method:**

Dentary surface perforations were examined by surface epi-illumination microscopy in tyrannosaurine and albertosaurine dinosaurs to characterize their anatomy. Fish lateral lines were examined as potentially comparable structures.

**Result:**

In contrast to the subsurface vascular bifurcation noted in tyrannosaurines (which lack a lateral dentary surface groove), the area subjacent to the apertures in albertosaurine grooves has the appearance of an expanded chamber. That appearance seemed to be indistinguishable from the lateral line of fish.

**Conclusion:**

Dentary groove apertures in certain tyrannosaurid lines (specifically albertosaurines) not only have a unique appearance, but one with significant functional and behavior implications. The appearance of the perforations in the dentary groove of albertosaurines mirrors that previously noted only with specialized neurologic structures accommodating derived sensory functions, as seen in the lateral line of fish. The possibility that this specialized morphology could also represent a unique function in albertosaurine theropods for interacting with the environment or facilitating prey acquisition cannot be ignored. It is suggested that these expanded chambers function in perceiving and aligning the body relative to the direction of wind, perhaps a Cretaceous analogue of the contemporary midwestern weathervane.

## Introduction

The ratio of the relative size of the olfactory lobes of the brain compared to the greatest longitudinal linear measurement of the cerebral hemispheres has been utilized as a measure of sensory, or at least of olfactory function, in a wide variety of predatory and scavenging species of birds and dinosaurs [1–4]. Zelinitsky *et*. *al* (2011) surveyed 157 taxa of birds and theropod dinosaurs using this method to assess the degree of olfactory capability in these animals. While the conclusions remain somewhat controversial, because there is still uncertainty with regard to the ratio of olfactory bulb size to cerebrum length in *Tyrannosaurus rex*, the available evidence has been interpreted as sufficient to predict a scavenging lifestyle for this animal [5, 6]. Zelinitsky *et al*. (2011) also suggest the same habits for Albertosaurines even though their olfactory ratios are equally uncertain. Because of this lack of definitive information, it is desirable to generate an additional standard by which the olfactory sensory capabilities of tyrannosaurids and albertosaurines can be measured.

In the absence of visual cues, predators most commonly pursue carrion or living prey [3] by using olfaction to identify the food source. However, the localization of prey or carrion by such methods, is more complex than is spotting it visually. These feeding efforts might also involve special sensory organs. This is the hypothesis examined in this study, which characterizes mandibular groves in at least in some theropods. To date, no other clearcut function has been identified for these structures although several possibilities have been proposed. Vertebrates depend on a typical set of five sensory modalities, although some species have evolved the ability to detect other environmentally-pertinent information [7, 8]. At times these enhancements have even resulted in the modification of sense organs for other purposes (Table 1).

**Table 1:** Sensory organ modification.

Sensation	Organ	Organism	Reference
Sound detection	Vibrissae	Rat	[9]
		Pinniped	[10–12]
Electroreceptors	Ampullae of Lorenzini	Sharks	[13, 14]
Magneto-detection	Olfactory	Fish	[15–19]
	Beak	Bird	[17, 20, 21]
	Ocular	Bird	[22]
	Pineal	Bird	[17, 23]
	Olfactory bulb	Bird	[24, 25]
Infra-red radiation	Pits	Buprestid *Melanophilia acuminate*	[26]
	Labial scales	Pythons and boas	[27, 28]
Thermal detection	Pits	Vipers	[29]
	Tentacles	Snake *Erpeton tentaculatus*	[30]
Chemical detection	Strigolactone-sensor	Witchweed *Striga hermonthica*	[31]
Oxygen detection	Neuro-epithelial cells	Mice	[32]
Respiration detection	Acid-base	Japanese sea catfish *Plotosus japanicus*	[33]
Mechanoreceptors	Scale sensilla	Reptiles	[34]
Fluid movement	Lateral line	Fish and amphibians	[35]

Vertebrates have evolved various specialized receptors to detect light, mechanical and chemical stimuli, temperature variation and tissue damage (nocioceptors) [36]. An example of this degree of specialization of sensitivity is exemplified by rat vibrissae, which respond to sound stimulation at specific frequencies [9]. Vibrissae of other mammals such as Pinnipeds are particularly sensitive to waves and their movement patterns. These structures can even track different types of hydrodynamic trails by detection of the frequencies generated by the resultant wave motion [11]. Modern crocodilians have a unique system of integumentary sensory organs that are used to detect water temperature, salinity, and pressure variation. Other examples include the pressure-sensing lateral line system that has many different morphologies. Various forms of this system have been recognized to date in fish and aquatic amphibians [35]. This is the sensory mode that is pertinent to the current observations.

The lateral line system has previously been demonstrated to detect the direction of water movement. It responds to low frequency mechanical signals and is utilized in detection of predators or prey, obstacles, is used to facilitate fish schooling behavior, and in sexual communication [37, 38]. Placement of these sensory organs in grooves restricts the impinging pressure waves to arrive at right angles to the body surface, thus protecting them from the effect of the movement of the animal. These grooves were originally perceived as mucous-secreting organs, until Leydig discovered that the grooves contained neuromasts in 1850 [39]. The lateral line system is structurally homologous to the inner ear of tetrapods [40], with analogous phenomena noted in cephalopods (e.g., cuttlefish *Sepia officinalis*, the brief squid *Lolliguncula brevus*) and aquatic mammals (e.g., the whiskers of the manatee *Trichechus* and harbor seals *Phoca vitulina*) [41].

The lateral line in fish, as characterized in *Amia calva* and *Atractosteus spatula*, is recognized as a series of grooves [lateral line canals] traversing the surface of the mandible and upper skull [42]. Within the grooves are pits containing multiple well-defined, circular and elliptical spaces with sub-surface expansions [42]. These appear to represent a “housing” for the cupula, the jelly-like sheath encapsulating a cluster of sensory and support cells [39]. Interest in the role of dentary foramen [43], speculation as to presence of a dermal sensory system in other Mesozoic reptiles [44,45], characterization of the microscopic appearance of the lateral line in fish [42], and recognition of a previously undescribed, but similar structure in the dentary of albertosaurines [46] stimulated this report.

## Methods

The dentaries of tyrannosaurines and BMR P2002.4.1 and other albertosaurines (Table 2) were examined macroscopically and with epi-illumination microscopy (AD7013MZT Dino-Lite, Microscope, The Microscope Store, LLC, 1222 McDowell Avenue, Roanoke, VA 24012) to describe a macroscopically recognized, but apparently largely unexplored character, a lateral groove and its perforations.

**Table 2:** Phylogenetic dentary groove distribution examined in tyrannosaurids.

Groove status	Genus	Collection number
Present	*Gorgosaurus*	TMP 86.205.1
		TMP 99.55.170
		TMP 82.28.1
		TMP 86.144.1 juvenile
		TMP 86.49.29
		TMP 1983.36.134
		TMP 1992.36.749
		TMP 1991.036.0500
		BHI #126850
	*Albertosaurus*	TMP 1967.9.164
		TMP 2003.045.0076
		AMNH 5664
Absent	*Tyrannosaurus*	TMP 1981.006.0001
		LACM 238471 juvenile
		AMNH 5027
		NMNH Peck rex
	*Daspletosaurus*	TMP 75.11.3
		TMP 2002.12.101
		TMP 2010.5.7
		TMP 87.48.4
		TMP 1981.003.0006
		TMP 94.143.01
		TMP 2001.036.0001
	*Zhuchengtyrannus*	ZCDM V0031

AMNH–American Museum of Natural History

BHI–Black Hills Institute. Specimen currently curated at Indianapolis Children’s Museum

LACM–Los Angeles County Museum

NMNH–National Museum of Natural History

TMP–Royal Tyrrell Museum

ZCDM—Zhucheng Dinosaur Museum

The phylogenetic data base was augmented with additional specimens from the Royal Tyrrell Museum. The groove is distinguished from the folded appearance seen in the pliosaurid *Megacephalosaurus eulerti* [47]. The opposing edges of the groove are in the same two-dimensional plane, in contrast to folds which are non-planar. The lateral line correlates were recognized on the basis of pits containing multiple well-defined, circular and elliptical spaces with sub-surface expansions [42]. Penetrating vascular channels were recognized on the basis of smooth boundaries and internal bifurcation [42]. The groove and dentary perforations in Jane were compared to those noted in *Amia calva* and *Atractosteus spatula*.

## Results

**Fig 1:**
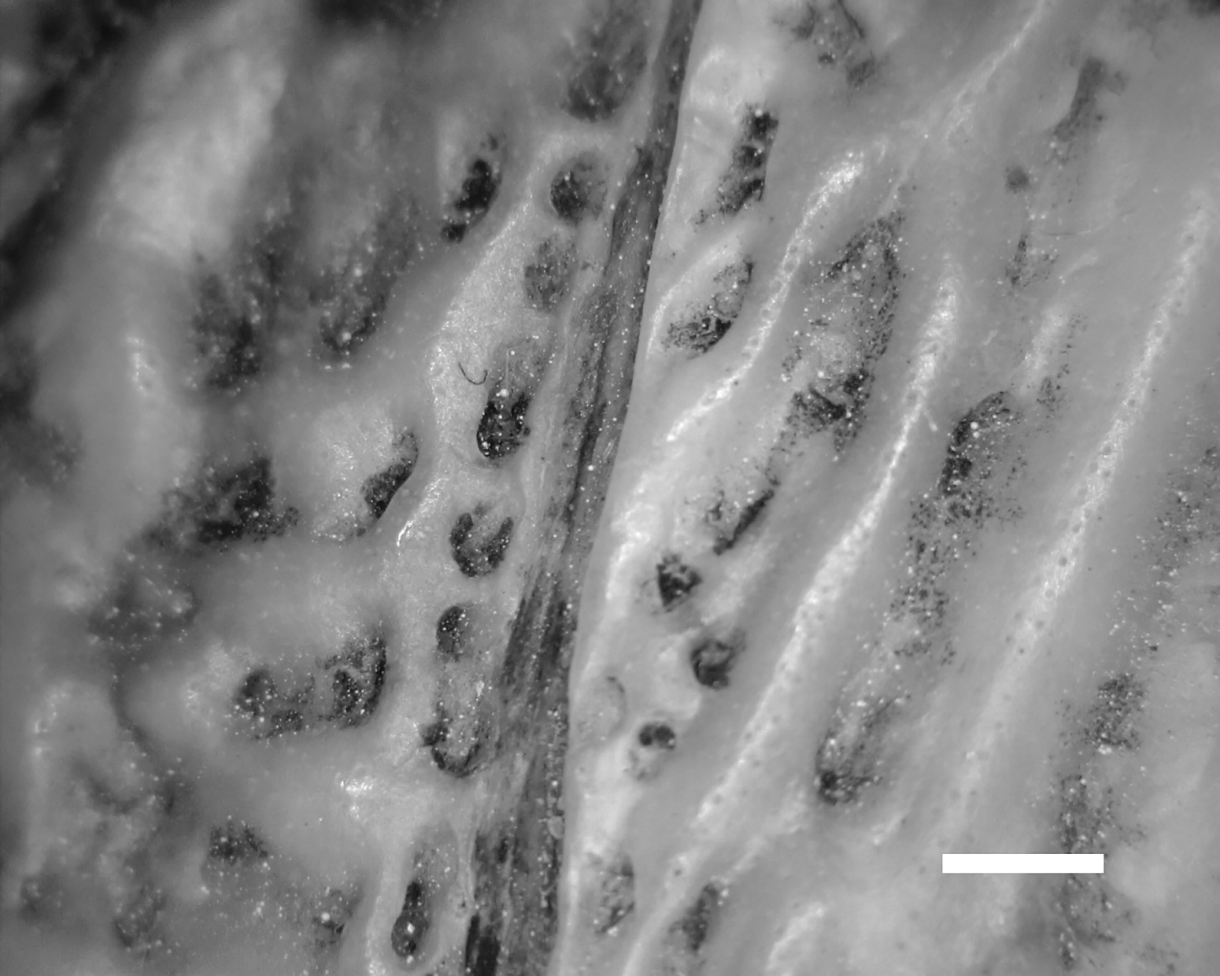
En face view of lateral lines of *Amia calva* skull. Multiple well-defined circular and irregular ellipse “pores” with expansile bases. Bar = 0.5 mm.

**Fig 2:**
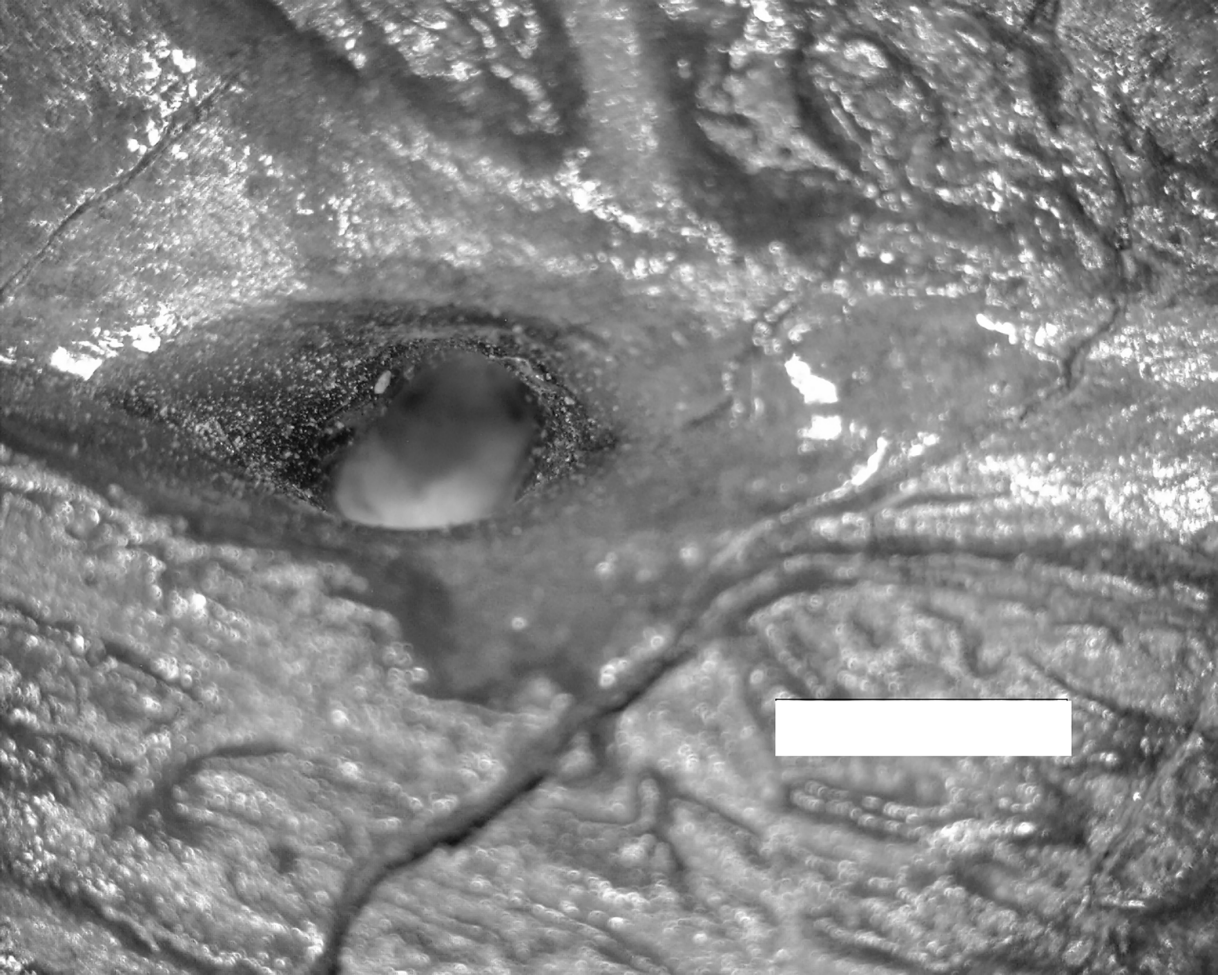
En face view of lateral aspect of dentary of tyrannosaur Jane. “Pores” are limited in distribution to a lateral mandibular groove, and do not bifurcate at the base. Bar = 2 mm.

Dentary grooves were present in all of the albertosaurines examined (Table 2). The structures in the groove of the albertosaurines as well as the grooves themselves mirror the osseous manifestations of the lateral lines seen in fish (Fig 1) [42]. The groove perforations differ from those [openings that are not in grooves] in *Tyrannosaurus rex* dentaries, which clearly have penetrating vascular channels. The latter are recognizable on the basis of their smooth boundaries and internal bifurcation [42]. The tyrannosaur known as Jane, (specimen number) is the skeleton of a juvenile dinosaur on exhibit at the Burpee Museum of Natural History in Rockford, Illinois. This individual is approximately eleven years of age and is one of the most complete among tryannosaurs, with about 85% of the bones represented. Jane’s dentary has penetrating channels, but these differ in two major aspects from those seen in *T*. *rex*. They are limited in distribution to the lateral mandibular groove, and they do not bifurcate at the base (Fig 2). Instead, the individual channels terminate in an expanded base.

## Discussion

Determining the location of prey is as important as identifying its presence somewhere in the environment [48]. Olfaction simply identifies the presence of a potential prey item. It must be combined with other sensory input for successful prey acquisition [2]. Locating the source of the stream of chemicals that animals release into the environment stimulates specific behaviors. The usual response of the predator is movement toward the greatest concentration of odorant. Rather than follow the odor plume, the pursuing animal flies directly upwind [e.g., moths, oriental fruit flies, tsetse flies *Glossina* spp.] [49,50]. When the odorant is no longer detected, they move back and forth lateral to the wind, a term referred to as “casting about,” until they detect it again, at which point they again head upwind. [51]. However, such movement increases the possibility that the prey will see the predator and flee. Additionally, the technique is not instantaneous. Wind velocity must be above a certain level for a predator to detect prey without the prey taking notice [52]. It must exceed 3 km/hr in moths *Spodoptera litura*, 3.6 km/hr in tsetse flies and 10 km/hr in dogs [48–50].

Environmental effects on the path and concentration of an odor plume are significant. Change in wind direction, of course, makes odor plumes inconsistent [48]. On sunny days, forest temperatures are comparatively cool. The specific heat of trees and water content are higher than those of soil. Heat is lost through transpiration, and less heat is available due to blocked sunlight. At night, forests are warmer because the canopy retards loss of long-wave radiation. Enhanced capability to perceive wind and smell increases the efficiency of locating prey within a forest. Turbulence from gusts in a forest sub-canopy makes odor plumes dissipate more rapidly, making them harder to follow. Therefore, predators in forested environments require greater efficiency to detect and maintain pursuit of a scent, to be effective in acquiring the prey item, in contrast to animals hunting in savannah environments [52,53]. Tree row spacing also has a major effect on wind speeds. When trees are spaced at eight by eight meter intervals, wind speed is reduced by 54%; at six by six meter intervals, 71%; and at four by four meter intervals, 84% [53]. Hunting efficiency would be increased by any adaptation that reduces casting-about time, because this would minimize the chance of odorant plume dissipation. This reduction in the length of time to identify the location of prey is especially pertinent in more densely forested environments. Such environmental characteristics may have been a factor in the evolution of this new character in albertosaurines (see below).

A structure, apparently not previously described in tyrannosaurids, is present in the albertosaurines. There has been limited previous recognition of the theropod mandibular groove [46, 53, 54]. Contrary to the assumption by Carr et al. [55], this structure is limited in distribution to albertosaurines [53]. While another type of perforation is observed in tyrannosaurines, grooves are conspicuously absent [46, 56]. Most authors have been uncritical of this feature, referring to it either as simply as a groove (e.g. 55, Figs 1 and 2), as a neurovascular groove (e.g. figure 1B1 of Brusatte et al.) [57], as a dentary sulcus, a mental groove (e.g, [58]) or have illustrated or figured the feature with no description whatsoever (e.g., [59], Fig 2 and 15). The groove has been assumed to have a vascular function without critical examination of the morphology of the fenestrae in the groove. Recently, Carr et al. [55], repeated Ford’s 2015 suggestion [43] that dentary fenestra have a tactile stimulatory function. This represents an oversimplification of what appears to be a previously undescribed sensory capability in dinosaurs.

A similar structure has been reported in the tetanuran *Duriavenator hesperis*, wherein it was thought to be a “well-defined groove for nutritional foramina” [37, p. 138]. It is actually analogous to the lateral line of fish, with the same ontogenetic implications [38], and a similar function is suggested. Olfaction alerts the tyrannosaurid to the presence of potential food. The system represented by the dentary groove contained pits which identify the direction from whence the aroma of interest arises. Exposed in open gape, they relate to wind direction rather than smell, facilitating targeting of carrion or prey. Evans [26] described sensory pits in the Buprestid *Melanophilia acuminate*. The attributed function is sensory, detecting infra-red radiation, allowing orientation of this insect to fires, at a range of as much as 60–100 miles. In the same manner, the pits in BMR P2002.4.1 would appear to have this behavioral implication, identifying wind direction.

Pressure-sensitive organs have been previously recognized in reptiles. Melanin-pigmented dome pressure receptors [also known as integumentary sensory organs] are present on virtually all of the scales of crocodilians and gharials, although they are more limited in distribution in alligators and caimans [36]. They represent a high resolution mechanosensory system, present one-to-one in postcranial scales, but with multiple representations on cranial scales. There is an ellipsoidal [postcranial] or spherical [jaw and neck] region just underlying the modified epidermis. The cranial receptors detect surface waves, allowing orientation towards disturbances of the water-air interface [36, 60, 61]. This represents transformation by the ancestor of the crocodilians of the ancestral diffused sensory system of the skin [still present in all other vertebrates], similarly innervated by branches of the trigeminal nerve [36,62]. The primitive diffused sensory system was modified in the crocodilian lineage into an array of discrete, micro-organs innervated by multiple pools of sensory neurons. The presence of anatomically similar surface phenomena to those recognized in the lateral line in fish is parsimonious with analogous function.

The utility of this sense organ would be in determining the direction of the wind, and therefore more accurately pinpointing the origin of a detected smell. The presence of the system on both sides of the head would allow the organ to act as a paired organ system, with differences in perception between the left and right sides allowing the animal to orient itself. In a way analogous to binocular vision, overlapping fields of smell perception could be used to orient the body relative to wind direction. This seems to be analogous to the infrared-sensitive labial scales in pythons and boa (Table 1) [26].

Further supporting the perspective that abertosaurines had a sensory mechanism that appears lacking in tyrannosaurines is evidence suggestive of differential sensory processing. There appears to be a difference between the brains of tyrannosaurines and of albertosaurines or at least *Gorgosaurus* [3]. *Gorgosaurus* as studied has widely separate ophthalmic and maxillomandibular canals [compared to those of three *Tyrannosaurus rex* skulls] and apparently greater diploic vein drainage. Full assessment of genus/family-related brain differences awaits preparation of subtraction images, that is, those that can adjust for size, and overlaying the 3D brain images to identify what areas are more or less represented. Witmer and Ridley’s [3] landmark study is a start of a process to understand anatomical contributions to behavior, one continued by Brusatte et al. [61] and further advanced by the current analysis of albertosaurine sensory modalities.

Our understanding of the neurovascular system in the Mesozoic is in its infancy. We hypothesized [46] that *Tyrannosaurus* and *Daspletosaurus*, lacking this system, differed from albertosaurines in food acquisition behaviors. Tyrannosaurines may have depended on the purview provided by their heights and visual identification of food sources, while albertosaurines may have been more dependent on localization by interpretation of wind direction. *Tyrannosaurus* clearly could position its head at a greater height above ground than the albertosaurines, but what of *Daspletosaurus*? Though similar in size to *Gorgosaurus*, but more massively built [5], and sharing at least some dietary habits, Holtz [63] suggested that *Daspletosaurus* predominantly occupied a more southern habitat than *Gorgosaurus*. One consideration is that floral variation [e.g., height of trees in that location] increased the importance of sighting prey in the environments occupied by *Daspletosaurus*, reducing the evolutionary advantage of a non-visual system for localizing smells.
